# Antitrypanosomal activity of hydromethanol extract of leaves of *Cymbopogon citratus* and seeds of *Lepidium sativum*: *in-vivo* mice model

**DOI:** 10.1186/s12906-021-03449-1

**Published:** 2021-11-27

**Authors:** Ayechew Yetayeh Emiru, Eyasu Makonnen, Fikru Regassa, Fekadu Regassa, Takele Beyene Tufa

**Affiliations:** 1 School of Veterinary Medicine, Woldia University, P.O. Box 400, Woldia, Ethiopia; 2 College of Health Sciences, Addis Ababa University, Addis Ababa, Ethiopia; 3 College of Veterinary Medicine and Agriculture, Addis Ababa University, Bishoftu, Ethiopia; 4 Department of Farm Animal Health, Faculty of Veterinary Medicine, Utrecht University, Utrecht, The Netherlands

**Keywords:** Antitrypanosomal activity, *Cymbopogon citratus*, *Lepidium sativum*, *Trypanosoma congolense*

## Abstract

**Background:**

Trypanosomiasis is one of the neglected tropical diseases of both humans and animals which decreases their productivity and causes death in the worst scenario. Unavailability of vaccines, the low therapeutic index of trypanocidal drugs, and the development of resistance lead to the need for research focused on developing alternative treatment options especially from medicinal plants. The present study was aimed to investigate antitrypanosomal activities of leaves of *Cymbopogon citratus* and seeds of *Lepidium sativum* in *in-vivo* mice model.

**Methods:**

The plant extracts were prepared by maceration using 80% methanol and reconstituted with 10% dimethyl sulfoxide (DMSO) to have the desired concentration. The test doses were adjusted to 100, 200 and 400 mg/kg based on the toxicity profile. The plants extracts were administered to the respective groups of mice after the 12^th^ day of field isolate *T. congolense* inoculation for seven consecutive days. The level of parasitemia, bodyweight, packed cell volume (PCV), and differential white blood cell counts were measured.

**Results:**

The* in -vivo* test results revealed that both plant extracts had dose-dependent antitrypanosomal activity. Both crude extracts showed a significant reduction in parasite load (*P* < 0.05), increased or prevent the fall of PCV value (P < 0.05), decreased lymphocytosis and increased neutrophil counts (*p* < 0.05) and improved bodyweight but significant bodyweight increment (P < 0.05) was observed only in *C. citratus* treated mice compared to the negative and positive controls.

**Conclusion:**

The present study concluded that the crude extracts of leaves of *C. citratus* and seeds of *L. sativum* had antitrypanosomal effects. Both plants extracts reduced parasitemia level, prevented anemia and improved bodyweight of treated mice. Comparative results from all tested parameters showed that the best activities were observed with *C. citratus* treated groups of mice.

## Background

Trypanosomiasis is a protozoan disease caused by the genus trypanosome. The disease affects both animals and human beings. African animal trypanosomiasis causes severe economic losses in the livestock sector. Morbidity and/or mortality rates for African animal trypanosomiasis are influenced by an animal’s general health, as well as the strain and dose of the infecting organisms [1, 2].

Human trypanosomiasis is one of the 13 most neglected tropical diseases recognised by the world health organization. *Trypansoma cruzi* which was discovered in 1909 is the cause of American trypanosomiasis (Chagas disease) [3]. Human African trypanosomiasis (HAT) is common in sub-Saharan Africa. There are two forms of HAT; slow-progressing form, caused by *Trypanosoma brucei gambiense*, which is endemic in western and central Africa; and faster-progressing form, caused by *Trypanosoma brucei rhodesiense*, originate in eastern and southern Africa including Ethiopia [4, 5]. The severity of clinical presentations of the disease varies with those forms of the disease. Rhodesiense HAT is an acute disease that usually progresses to death within 6 months; whereas gambiense HAT has a more chronic progressive course with an average duration of 3 years [6].

The disease is considered to have an approximately 100% case fatality rate. Despite the lethality and the individual-level effect, the disease has enormous socio-economic impacts. As the disease affects mainly individuals of productive age and mostly being a chronic disease, it affects the income-generating capacity of the people, worsening the economic situation of impoverished societies resulting in sustainability of the poverty cycle in the neglected communities. It is also a stigmatizing disease, primarily because of the neuropsychological impairment entailed, and in many endemic areas, the presence of the disease is hidden and the patients are discriminated against or abandoned [6, 7].

Due to the unavailability of vaccines, control and prevention of trypanosomiasis rely on chemoprophylactic and/or curative trypanocidal therapies. Despite their toxicity, most trypanocidal drugs are developing resistance both in AAT and HAT [8–11].

These factors emphasize the need for research into more comprehensive, formidable and more affordable sources of trypanocidal agents. Medicinal plants have paramount importance in the discoveries and development of new alternative drugs. In Ethiopia, many plants are claimed to be useful as traditional remedies for the treatment of trypanosomiasis in different parts of the country [12]. *Cymbopogon citratus* (lemongrass) known by the Amharic name ‘Teji sar’ in Ethiopia, is used for the treatment of many infectious and non-infectious diseases including trypanosomiasis. In vitro study of citral, the main component of *Cymbopogon citratus,* revealed that the plant has antitrypanosomal activity [13]. *Lepidium sativum* ‘Fetto’ in Amharic is also among the plants claimed to have antiprotozoal activity and/or antitrypanosomal effect in particular. A single dose in vivo test reported by Al-Otaibi et al., [14] concluded that the plant extract had antitrypanosomal activity against *T. evansi*. Therefore, the present study was targeted to investigate the in vivo antitrypanosomal activity of crude extracts of leaves of *C. citratus* and seeds of *L. sativum* at a variable concentration.

## Materials and methods

### Experimental animals

Swiss albino mice aged 8–12 weeks and weighing 25–35 g were obtained from  the Ethiopian public health institute. Both male and female mice were used for the in vivo antitrypanosomal activity test but only female mice were used for acute toxicity study. Animals were housed in polypropylene cages six mice per cage and allowed free access to clean water ad libitum and feed (pellet). They were kept at room temperature (23^o^c) having 12 h day and night.

### Test organism

*Trypanosoma (Nannomonas) congolense* is the most prevalent and widespread pathogenic trypanosome in tropical Africa, being found in ruminants, pigs, dogs and other domestic animals throughout the tsetse belt [15]. In the mammalian bloodstream, *T. congolense* is a small trypanosome, shorter in length and without a noticeable undulating membrane. In the vector (tsetse fly), initially it develops and multiplies in the midgut; while infective metacyclics develop in the proboscis [16]. The field isolates of the organism were obtained from Ghibe valley known with a high prevalence of trypanosomiasis and tsetse fly infestation [17]. We had established a temporal field laboratory using generator power. A drop of blood was sampled from the ear vein of trypanosomiasis suspected animals using microscopic slides and examined under the microscope after adding the cover slip. Blood was collected from the jugular vein of trypanosomosis diseased animals using EDTA coated tubes which is then inoculated into mice which were then used as a donor to the experimental mice.

### Chemicals and equipment

#### Chemicals and solvents

Dimethyl sulfoxide (DMSO), Methanol, distilled water, and reference drugs (Diminazene aceturate, DA), modified phosphate buffered saline solution supplemented with glucose (PBSG) were used in the study.

#### Materials

Aluminium foils, cover slide, microscopic slide, mortar and pestle, digital weighing balance, diamond pencil, desiccator, Ethylenediaminetetraacetic acid (EDTA) coated syringe, heparinized capillary tube, Whatman No.1 filter paper, glove, haematocrit reader, microhematocrit centrifuge, oven, refrigerator, petridish, microscope, micropipettes, Rotary evaporator, sterile lancet, syringe 1 ml, cristaseal, spatula and flasks were employed in this study.

### Plant materials and test organism collection

The plant materials were collected according to a Preliminary Guide to Plant Collection, Identification and Herbarium Techniques, National Herbarium of the Addis Ababa University. Plant materials were collected from Debre Elias district of the East Gojam zone. The district is located around 341 km northwest of the capital city of Ethiopia, Addis Ababa. The mean annual temperature of the district ranges from 18 to 27 °C and receives a mean annual rainfall of 1150 mm with an altitude that ranges from 800 to 2200 m above sea level [18]. Whereas the test organism, *Trypanosoma congolense* was isolated from the Ghibe valley area of the Borer Tade'le peasant association of Abeshige district, Gurage Zone of the South Nations and Nationalities People Region.

### Laboratory where the experiment was carried out

The experiment was conducted in the Veterinary Pharmacology and Toxicology Laboratory of the College of Veterinary Medicine and Agriculture, Addis Ababa University, Bishoftu, Ethiopia.

### Study design

The randomized experimental design was employed in which the experimental animals were assigned to the control or experimental group and also between different experimental subgroups randomly.

### Methods

#### Pre-extraction preparation

Samples of the plant were authenticated by the national herbarium of Addis Ababa University College of Natural Science. The identification was done by Mr. Melaku Wondaferash (Botanist), and the report contained the local name, botanical name and its family; Teji sar- *Cymbopogon citratus* (DC.) Stapf- Poaceae and Fetto- *Lepidium Sativum L*.- Brassicaceae, and samples were deposited with specified voucher numbers (AY1 and AY3) respectively.

The plants were planted in the Garden of toxic and medicinal plants established by the corresponding author of this paper in 2016 at the College of Veterinary Medicine and Agriculture, Addis Ababa University, Bishoftu for future use.

After collection, the plant materials were washed with tap water to remove unnecessary particles, dried under shade, and grounded mechanically. The materials were sieved and weighed before subjected to extraction procedures.

#### Crude extract preparation

The plant materials were extracted by maceration technique using 80% Methanol (LOBA CHEMIE PVT LTD) through mixing the grinded and weighted plant material with 80% Methanol in 1:7 ratios. After 72 h maceration with regular shaking, the mixtures were strained using a strainer to remove solids and further filtered with Whatman filter paper No 1. The filtered solutions were evaporated using Rota vapour (BUCHI Rotavapor R-200) to remove the solvent to the acceptable level. Remnants from the Rota vapour were poured into petridishes and put in a dry oven at a temperature of 40 °C to remove the remained solvent. The prepared solid extracts were stored in a desiccator until the experimental procedures were conducted.

#### Acute toxicity test

The acute toxicity study was conducted according to the Organization for Economic Co-operation and Development (OECD), 2001 guideline fixed-dose (2000 mg/kg) toxicity test. A group of six female albino mice were fasted from food but not water for 4 h before administration and 2 h after oral administration of the test extract. The mice were critically followed continuously for 1 h after administration of the extracts; intermittently for 4 h over a period of 24 h for death, gross behavioural changes and other signs of toxicity. The follow-up continues for 14 days post-treatment [19].

#### Experimental mice inoculation

The donor mice were infected with field isolates of *T. congolense* directly from trypanosome infected cattle at the Ghibe valley. After 12 days of infection, the donor mice reached its peak level of parasitemia. Blood was collected from donor mice by cardiac puncture and/or drawing through tail veins after mice were anaesthetised using chloroform. Collected blood was diluted with phosphate buffered saline (PBS) to increase the volume of the inoculum. Healthy mice were injected intraperitoneally with 0.2 ml of the inoculum containing 10^7^ parasites per millilitre approximately [20]. After 9–11 days post- infection the experimental mice were tested for the development of parasitemia, and only positive mice were drawn into the experimental groups.

#### Dose adjustment, grouping of mice and administration of the plant extracts

Three test doses were adjusted as 100 mg/kg, 200 mg/kg and 400 mg/kg based on the toxicity profile of the extracts. The dried and weighted plant extracts were reconstituted with 10% DMSO to have intended concentrations. A total of 60 mice were then grouped into nine groups having six mice per group (CC100, CC200, CC400, LS100, LS200, LS400, DA3.5, DA28, NC and UU). CC100, CC200 and CC400 are groups of mice treated with 100, 200 and 400 mg/kg of *C. citratus* extract, respectively. LS100, LS200 and LS400 were 100, 200 and 400 mg/kg *L. sativum* extract treated mice groups. DA3.5 and DA28 were positive controls taking 3.5 mg/kg and 28 mg/kg standard drug DA [20], respectively. Whereas the NC group was the negative control infected with the parasite but treated only with 1 ml of 10% DMSO (vehicle). The UU group was uninfected untreated healthy mice used as a reference. The treatment was started on day 12 post infection. The extracts were administered every morning for seven consecutive days. The control groups (DA3.5, DA28 and NC) were treated once on the first day of the above-mentioned doses. All treatments were administered via the intraperitoneal route.

#### Determination of parasitaemia and body weight

Parasitaemia was monitored every other day starting from the first day of treatment and continues until the 14th day. These were done by microscopic examination of blood obtained from the tail of each mouse. The tail was cut to extrude blood, and a drop of blood was placed on a microscope slide and a wet smear was prepared by covering the drop by cover slips. The smears were examined microscopically at 400X total magnification (camera aided Olympus microscope). The degree of parasitaemia was determined using the “Rapid Matching” method of Herbert and Lumsden. Smears were prepared in triplicates from each animal and the mean value of slide counts was taken per sample examined microscopically. Logarithm values of these counts were obtained by matching with the table and the charts given by Herbert and Lumsden [21].

The body weight (in gram) of each mouse in all groups was taken on the day of treatment was commenced (day 0) and every other day (on Days 2, 4, 6, 8, 10, 12 and 14) up to the 14th day.

#### Determination of packed cell volume (PCV) and differential white blood cell count

PCV was measured to predict the effectiveness of the test extracts in preventing hemolysis resulting from increasing parasitaemia associated with trypanosomiasis. It was monitored on Days 0, 7 and 14. Blood was collected from the tail of each mouse in heparinized micro haematocrit capillary tubes filled up to 3/4th of their length. The tubes were then sealed immediately with crystal seal and centrifuged in a micro haematocrit centrifuge (Hawksley micro haematocrit centrifuge, England) for 5 min. After centrifugation, the packed cell volume was measured using a haematocrit reader (Hawksley micro haematocrit reader, England). The effect of extracts in improving PCV of treated animals was compared with the controls.

The white blood cell count (WBC) was determined on thin blood films stained with Giemsa stain obtained from each mouse on the 14th day post extract administration.

### Data analysis

Data analysis was performed using Statistical Package for Social Science (SPSS) version 20. Data were expressed as mean ± standard error of the mean. One way ANOVA followed by Tukey’s multiple comparison tests was performed to determine statistical significance. *P* values less than 0.05 were considered significant.

## Results

### Percent yield of plant extracts

The test plants were weighed before and after the extraction process and the percentage yields were calculated. The result showed that hydromethanol extraction produced 15.03% yield for the plant *C. citratus* leaves and 16.48% from the seeds of *L. sativum*.

### Acute toxicity test

The acute toxicity test results showed that all mice that took both groups of the extract did not show any observable sign of toxicity at a dose of 2000 mg/kg.

### The effect of plant extracts on parasitemia level

#### Effect of 80% methanol extract of leaves of *Cymbopogon citratus* on the level of parasitemia

The result showed that 80% methanol extract of *C. citratus* reduced the level of parasitemia in mice treated with different concentrations of the extract. The onset of the decrement in the level of parasitemia varied with concentrations. At a dose of 100 mg/kg, a reduction started to be observed on the 8th day; a statistically significant reduction was, however, observed on the 10th day (*P* < 0.05) compared with the negative control and standard drug (3.5 mg/kg). At a dose of 200 mg/kg, the reduction in parasite load started to be observed on day 6 although a significant reduction was observed on the 10th day (*P* < 0.05) compared with the negative control and standard drug (3.5 mg/kg). The highest dose (400 mg/kg) reduced the level of parasitemia starting from the 6th day and significantly reduced along with day14, (P < 0.05) compared with those of negative control and at both doses (3.5 mg/kg and 28 mg/kg) of the standard drug (Table 1).

**Table 1:** Effect of 80% methanol extract of *Cymbopogon citratus* leaves on the level of parasitemia (expressed as mean ± SEM; n = 6)

Group of mice	Log value of parasitemia/ ml							
	D0	D2	D4	D6	D8	D10	D12	D14
CC-100	6.3 ± 0.11	7.5 ± 0.10	7.60 ± 0.13	8.0 ± 0.17	7.80 ± 0.21	7.50 ± 0.09^ab^	7.35 ± 0.05	7.05 ± 0.05
CC-200	7.1 ± 0.46	7.8 ± 0.40	8.20 ± 0.31	8.0 ± 0.09	7.68 ± 0.20	7.40 ± 0.10^ab^	7.13 ± 0.04	6.80 ± 0.05
CC-400	6.7 ± 0.42	6.83 ± 0.40^a^	7.93 ± 0.23	7.5 ± 0.28	6.85 ± 0.06^ab^	5.60 ± 0.06 ^abc^	3.60 ± 1.13^abc^	1.80 ± 1.13^abc^
DA-3.5	7.38 ± 0.18	7.73 ± 0.18	8.10 ± 0.18	7.93 ± 0.32	8.16 ± 0.15	8.23 ± 0.20	8.26 ± 0.21	8.53 ± 0.13
DA-28	7.8 ± 0.20	5.53 ± 0.05	5.96 ± 0.29	7.35 ± 0.14	7.46 ± 0.07	7.80 ± 0.21	8.10 ± 0.12	8.40 ± 0.10
NC	7.2 ± 0.13	7.95 ± 0.06	7.65 ± 0.20	8.17 ± 0.10	8.25 ± 0.25	8.55 ± 0.06	8.55 ± 0.06	8.70 ± 0.13

D = day; D0 = the day treatment commenced; SEM = standard error of mean; CC = *Cymbopogon citratus*; NC = negative control taking 1 ml 10% DMSO; 100, 200, 400, 3.5, and 28 are doses in mg/kg; DA = Diminazene aceturate; All superscripts indicate significance at p < 0.05 (a = compared to negative control; b = compared to 3.5 mg/kg DA; c = compared to 28 mg/kg DA

#### Effect of 80% methanol extract of seeds of *Lepidium sativum* on the level of parasitemia

Even though there was a fluctuation of values, infected mice treated with *L. sativum* extract showed a reduction in parasite load from days 2 to 8 as shown in Table 2. After the 8th day, the level of parasitemia increased as a relapse but the rate of increment was less than that of the negative control. At 100 and 200 mg/kg doses of the extract, a significant reduction was observed on the 8th day (*P* < 0.05) compared with negative control (1 ml 10% DMSO) and standard drug of DA (3.5 mg/kg). At 400 mg/kg, the extract showed a significant reduction from days 6 to 14 compared with the negative control and DA (3.5 mg/kg); while a significant reduction was observed only on the 14th day compared with DA (28 mg/kg).

**Table 2:** Effect of 80% methanol extract of seeds of *L. sativum* on the level of parasitemia (expressed as mean ± SEM, n = 6)

Group of mice	Log value of parasitemia/ ml							
	D0	D2	D4	D6	D8	D10	D12	D14
LS-100	8.08 ± 0.08	7.98 ± 0.10	7.83 ± 0.12	7.61 ± 0.10	7.30 ± 0.08^ab^	7.85 ± 0.08^a^	8.13 ± 0.09	8.33 ± 0.04
LS-200	8.30 ± 0.08	8.33 ± 0.04	8.45 ± 0.04^a^	7.98 ± 0.03	7.80 ± 0.10^ab^	7.85 ± 0.02^a^	8.00 ± 0.06^a^	8.26 ± 0.05^a^
LS-400	6.90 ± 0.40	6.43 ± 0.46^ab^	7.36 ± 0.14	6.93 ± 0.40^ab^	7.43 ± 0.04^ab^	7.53 ± 0.02^ab^	7.73 ± 0.04^ab^	7.96 ± 0.05^abc^
DA-3.5	7.38 ± 0.18	7.73 ± 0.18	8.10 ± 0.18	7.93 ± 0.32	8.16 ± 0.21	8.23 ± 0.20	8.26 ± 0.21	8.53 ± 0.13
DA-28	7.80 ± 0.2	5.53 ± 0.05	5.96 ± 0.29	7.35 ± 0.14	7.46 ± 0.20	7.80 ± 0.21	8.10 ± 0.12	8.40 ± 0.10
NC	7.20 ± 0.13	7.95 ± 0.06	7.65 ± 0.20	8.17 ± 0.10	8.25 ± 0.06	8.55 ± 0.06	8.55 ± 0.06	8.70 ± 0.13

D = day; D0 = the day treatment commenced; SEM = standard error of mean; LS = *Lepidium sativum*; NC = negative control taking 1 ml 10% DMSO; 100, 200, 400, 3.5, and 28 are doses in mg/kg; DA = Diminazene aceturate; All superscripts indicate significance at *p* < 0.05 (a = compared to negative control; b = compared to 3.5 mg/kg DA; c = compared to 28 mg/kg DA

#### Comparison of parasitemia level between groups

**Fig. 1:**
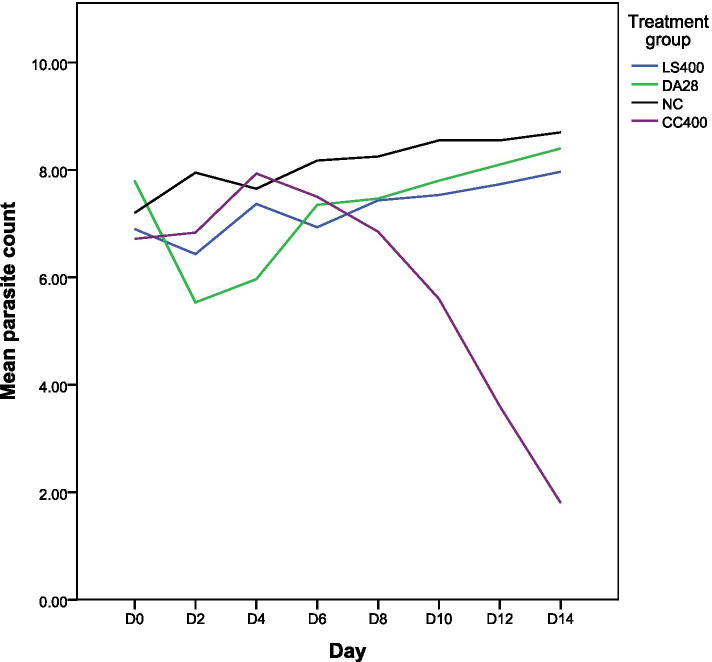
Comparison of the level of parasitemia treated with *L. sativum* and *C. citratus* with both control groups. Data are expressed as mean ± SEM. Mean parasitemia count = log value per ml of blood; CC = *C. citratus*; LS = *L. sativum*; NC = negative control (1 ml 10% DMSO); DA = Diminazene aceturate. 400 and 28 = are doses in mg/kg

As shown in Fig. 1, the comparative values of the highest doses of the extracts and control groups realized that methanol extract of *C. citratus* showed better activity compared with *L. sativum* extract and control groups.

### Effect of plant extracts on body weight

#### The effect of 80% methanol extracts of *Cymbopogon citratus* leaves on body weight

The present finding showed that extract improved the body weight of mice. As shown in Table 3, at all dose levels of the extract significantly increased (*P* < 0.05) bodyweight on the 12th and 14th days compared with the negative control and standard drug (DA) at both concentrations (3.5 and 28 mg/kg).

**Table 3:** Effect of *C. citratus* leaves extract on body weight (expressed as mean ± SEM; n = 6)

Group of mice	Body weight in grams							
	D0	D2	D4	D6	D8	D10	D12	D14
CC-100	33.6 ± .13	32.6 ± .13	35.0 ± .30	34.0 ± .51	35.6 ± .92	36.0 ± .48^c^	36.2 ± .44^c^	37.8 ± .43^abc^
CC-200	33.4 ± .77	32.6 ± .55	34.9 ± 1.28	33.3 ± .83	36.2 ± 1.33^c^	36.0 ± 1.15^c^	37.6 ± 2.34^abc^	36.8 ± 1.17^abc^
CC-400	32.8 ± 1.55	31.0 ± 1.42	31.5 ± 1.10	33.2 ± 1.24	33.5 ± .98	34.0 ± 1.01	34.5 ± .98^abc^	35.3 ± 1.17^abc^
DA-3.5	31.1 ± .78	31.9 ± .81	32.0 ± 1.01	32.3 ± 1.18	33.1 ± 1.06	33.1 ± .98	33.1 ± .93	33.2 ± .80
DA-28	31.5 ± .92	31.9 ± .93	30.9 ± 1.54	30.6 ± 1.36	31.2 ± 1.37	31.0 ± 1.28	30.9 ± 1.18	31.6 ± .93
NC	34.7 ± .74	34.3 ± .94	34.7 ± .84	34.4 ± .85	33.2 ± .70	33.0 ± .88	31.7 ± 1.13	31.0 ± .98

D = day; D0 = the day treatment commenced; SEM = standard error of mean; CC = *Cymbopogon citratus*; NC = negative control taking 1 ml 10% DMSO; 100, 200, 400, 3.5, and 28 are doses in mg/kg; DA = Diminazene aceturate; All superscripts indicate significance at *p* < 0.05 (a = compared to negative control; b = compared to 3.5 mg/kg DA; c = compared to 28 mg/kg DA

#### Effect of 80% methanol extracts of *Lepidium sativum* seeds on body weight of mice

As presented in Table 4, methanol extract of seeds of *L. sativum* increased the body weight of treated mice though all values were not statistically significant compared with both groups of controls.

**Table 4:** Effect of 80% methanol extract of *L. sativum* seeds on body weight (expressed as mean ± SEM, n = 6)

Group of mice	Body weight in grams							
	D0	D2	D4	D6	D8	D10	D12	D14
LS100	35.4 ± 1.5	34.7 ± 1.53	34.3 ± 1.86	35.1 ± 1.98	34.5 ± 1.72	34.5 ± 1.72	34.5 ± 1.74	35.0 ± 1.79
LS200	31.8 ± 0.38	33.9 ± 0.73	32.9 ± 0.81	32.4 ± 1.05	33.3 ± 0.85	33.3 ± 1.01	33.3 ± 1.15	34.6 ± 1.40
LS400	33.7 ± 0.61	32.1 ± 0.54	32.2 ± 0.67	32.3 ± 0.65	33.1 ± 0.67	33.6 ± 0.68	34.2 ± 0.69	35.2 ± 0.82
DA3.5	31.1 ± 0.78	31.9 ± 0.81	32.0 ± 1.01	32.3 ± 1.18	33.1 ± 1.06	33.1 ± 0.98	33.1 ± 0.93	33.2 ± 0.80
DA28	31.5 ± 0.92	31.9 ± 0.93	30.9 ± 1.54	30.6 ± 1.36	31.2 ± 1.37	31.0 ± 1.28	30.9 ± 1.18	31.6 ± 0.93
NC	34.7 ± 0.74	34.3 ± 0.94	34.7 ± 0.84	34.4 ± 0.85	33.2 ± 0.70	33.0 ± 0.88	31.7 ± 1.13	31.1 ± 0.98

D = day; D0 = the day treatment commenced; SEM = standard error of mean; LS = *Lepidium sativum*; NC = negative control taking 1 ml 10% DMSO; 100, 200, 400, 3.5, and 28 are doses in mg/kg; DA = Diminazene aceturate

### Effect of plant extracts on packed cell volume (PCV)

#### The effect of 80% methanol extract of *C. citratus* on packed cell volume (PCV)

**Fig. 2:**
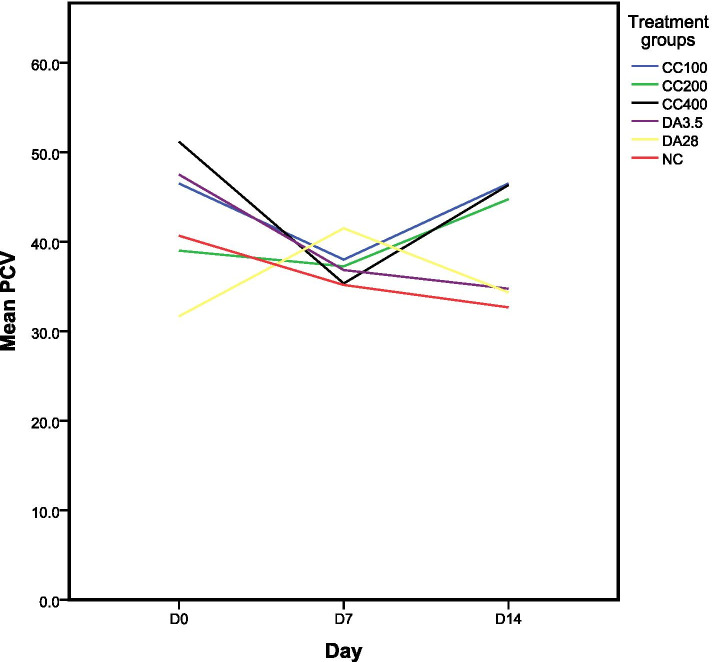
Effect of 80% methanol extract of *C. citratus* leaves on packed cell volume (expressed as mean ± SEM; *n* = 6).  D = day; D0 = the day treatment commenced; SEM = standard error of mean; CC = *Cymbopogon citratus*; NC = negative control taking 1 ml 10% DMSO; 100, 200, 400, 3.5, and 28 are doses in mg/kg; DA = Diminazene aceturate; PCV = packed cell volume.

Figure 2 showed a reduction in PCV values on day 7 though the rate of reduction was less than the negative control and DA (3.5 mg/kg). On the 14th day, all dose levels of the extract (100, 200, 400 mg/kg) significantly (*P* < 0.05) increased the PCV value compared to the negative control taking 1 ml 10% DMSO, and DA at doses of 3.5 mg/kg and 28 mg/kg.

#### Effect 80% methanol extract of *Lepidium sativum* seeds on packed cell volume (PCV)

**Fig. 3:**
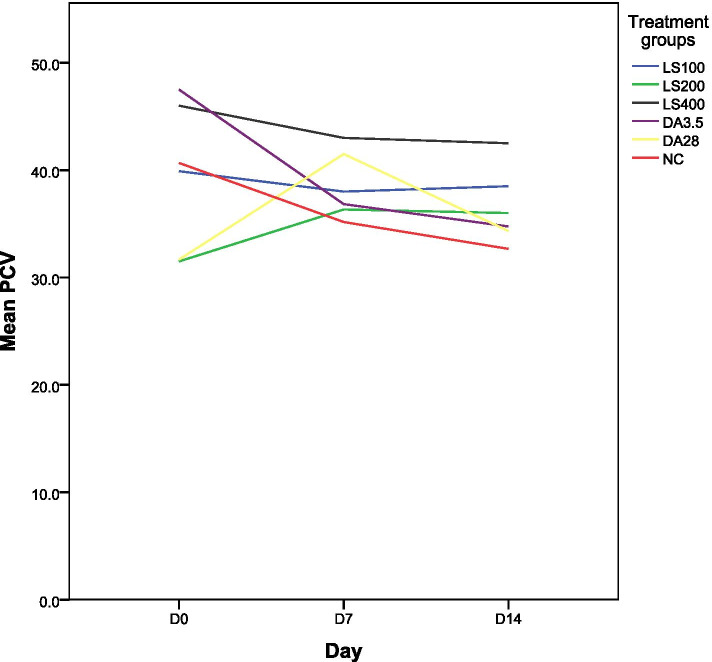
The effect of 80% methanol extract of *L. sativum* on packed cell volume (expressed as mean ± SEM; *n* = 6). D = day; D0 = the day treatment commenced; SEM = standard error of mean; LS = *Lepidium sativum*; NC = negative control taking 1 ml 10% DMSO; 100, 200, 400, 3.5, and 28 are doses in mg/kg; DA = Diminazene aceturate; PCV = packed cell volume

As shown in Fig. 3, *L. sativum* seeds extract increased the PCV value on day 7 at a concentration of 200 mg/kg though it was not statistically significant (*P* > 0.05). On the 14th day all dose levels of the extract significantly reduced (*P* < 0.05) rate of reduction in PCV value compared with all groups of controls.

### Effect of plant extracts on differential white blood cell counts

**Fig. 4:**
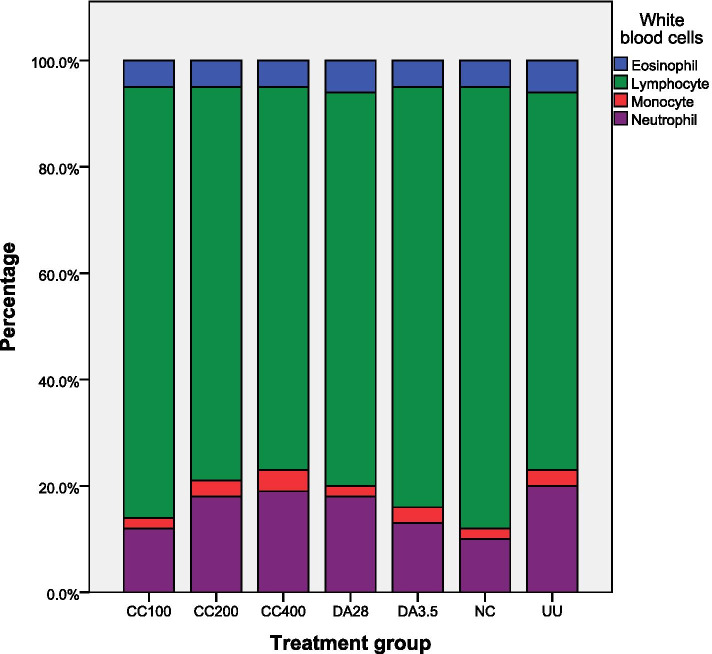
Effect of *C. citratus* leaves extract on differential white blood cell counts. CC = *Cymbopogon citratus*; NC = negative control taking 1 ml 10% DMSO; 100, 200, 400, 3.5, and 28 are doses in mg/kg; DA = Diminazene aceturate; UU = uninfected untreated group; PCV = packed cell volume

**Fig. 5:**
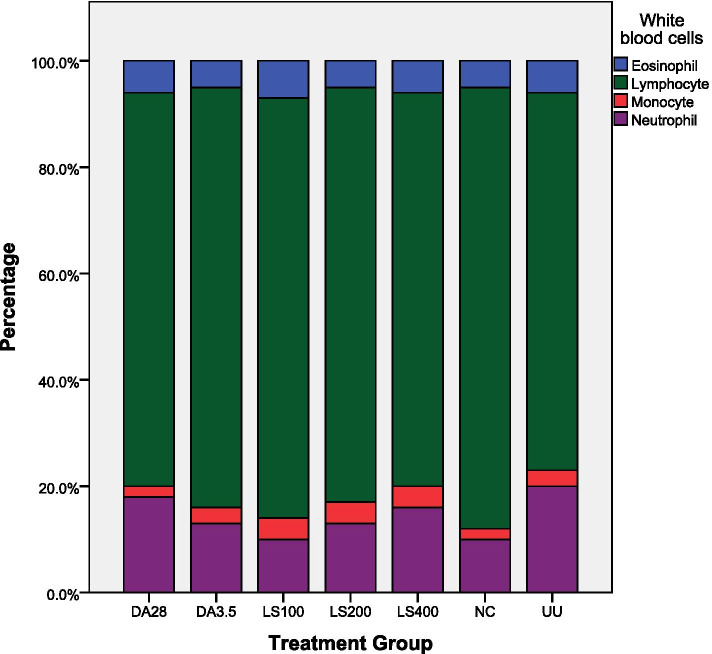
Effect of *L. sativum* seed extract on differential white blood cell count. LS = *Lepidium sativum*; NC = negative control taking 1 ml 10% DMSO; 100, 200, 400, 3.5, and 28 are doses in mg/kg; DA = Diminazene aceturate; UU = uninfected untreated group; PCV = packed cell volume

As shown in Figs. 4 and 5, the differential white blood cell counts showed that both plant extracts significantly decreased lymphocyte count and increased neutrophils at 200 and 400 mg/kg compared with infected-untreated and DA (3.5 mg/kg) groups. The highest percentage of lymphocytes was observed in mice that were infected with *T. congolense* but treated with 1 ml 10% DMSO (NC). Mice treated with 400 mg/kg *C. citratus* extract had a comparable proportion of cell counts with a pronounced increment in neutrophils compared with healthy mice (uninfected untreated). Both plant extracts showed statistical significance (*P* < 0.05) in reducing lymphocyte count and increasing the number of neutrophils and monocytes were observed only at their highest test dose (400 mg/kg) compared with negative and both doses of positive controls. But the values were statistically not significant compared with uninfected untreated healthy mice.

## Discussion

The main objective of this study was to evaluate the *in-vivo* antitrypanosomal effect of hydro-methanolic extracts of leaves of *Cymbopogon citratus* (lemongrass) and seeds of *Lepidium sativum* against field isolates of *T. congolense* in mice model. The results of the present study showed that both plants extract significantly reduced the parasite load though they did not completely clear parasites from the bloodstream of the infected mice. The reduced level of parasitemia observed by methanol extract of *C. citratus* leaves was in a dose-dependent manner. Administration of the extracts was continued for seven consecutive days but the reducing effect on the level of parasitemia was noticed starting on the 8th day and continued up to the 14th day. This implies that *C. citratus* extracts may have slow onset of action and/or the effect might be due to the cumulative effect of the repeated administration. Very pronounced anti-trypanosomal activity of *C. citratus* extract was observed on the 14th day at a dose of 400 mg/kg at which the mean logarithmic value of parasites per ml was less than 5.4, i.e., no trypanosome organism was observed in 20 fields of the microscopic slide [21]. At this concentration, significant effects (*P* < 0.05) were observed from days 10 to 14 compared with all controls (negative control, 1 ml 10% DMSO and positive control (DA at a dose of 3.5 and 28 mg/kg). These results are in line with the findings of a previous *in-vitro* study that reported a dose-dependent antitypanosomal activity of the plant [13]. The antitrypanosomal effect of the plant might be attributed to the major component citral and/or the additive or synergistic effects of other components.

Methanolic extract of *Lepidium sativum* seeds also had antitrypanosomal activity reducing the level of parasite load in mice infected with *T. congolense* in a dose -dependent manner. At 400 mg/kg of the extract, a reduction in parasitemia was observed on Day 6, thereafter the load slightly increased as a relapse. But the rate of increment in parasitemia level was significantly very low (*P* < 0.05) compared with that of the controls. The present study results are in agreement with those of the previous study by Al-Otaibi et al. [14], which reported antitrypanosomal activity of *L. sativum* against *T. evansi*. Unlike the previous study that used single dose and fixed administration and the follow up periods were only for 4 days, the present study tried to show the effect of the extract in variable dose ranges and longer follow-up period (14 days) by considering the undulating nature of the level of parasitemia in the body of animals. This resulted in broader insight into their antitrypanosomal activity. The antitrypanosomal activity of the extract might be attributed to its antioxidant and/or its immune-boosting properties [14].

The standard drug, diminazene aceturate (DA) was tested as a positive control at its recommended normal dose (3.5 mg/kg) and the highest dose (28 mg/kg). DA is one of the most frequently employed trypanocidal drugs used to treat animal trypanosomiasis. At a dose of 3.5 mg/kg the drug did not show a significant effect on the parasite load throughout the study period; the level of parasitemia continued to increase from D0 to D14 in the same way as the negative controls which were infected but treated with the vehicle (DMSO). The highest dose (28 mg/kg) of DA employed in the present study, however, significantly reduced parasitemia (*P* < 0.05) between days 2 and 4 compared with lower dose (3.5 mg/kg), all doses of both extracts and the negative control. Starting on day 6, the level of parasite count increased rapidly up to the end of the follow up period, i.e., day 14. These findings might suggest that the drug is at an alarming level of resistance. The results of the present study are in line with those of previous studies carried out on the development of drug resistance by trypanocidal agents [22–27].

In the present study, *Cymbopogon citratus* leaves showed better activity in reducing the parasite count than *Lepidium sativum* seeds. The antitrypanosomal activity of the plant extracts could also be deduced from their effect in improving body weight. Both extracts increased body weight throughout the study period. The weight gain in mice treated with all doses of *C. citratus* extract was statistically significant between days 12 and 14 compared with negative and both positive controls. Even though mice treated with *L. sativum* extract seems to increase their body weight, the values were not statistically significant. As AAT is associated with anaemia, decreased appetite and a rapid weight loss which progresses to extreme emaciation [28], the observed effect on body weight by the extracts can be associated with a reduction in parasite load in the bloodstream.

Severe anaemia is one of the causes of death associated with trypanosome infection. In the present study, the test extracts especially that of *C. citratus* significantly ameliorated the level of anaemia. Packed cell volume (PCV) improvement and prevention of further drop observed with both extracts mainly at the second week of treatment where parasitemia levels were very low could show antitrypanosomal effect of the extracts. The increment in PCV could be attributed to the reduction of the proliferating parasite load, neutralization of the toxic metabolites produced by trypanosomes or scavenging the trypanosome associated free radicals. DA at a dose of 3.5 mg/kg was not able to improve the PCV of infected mice, but it sharply increased the PCV value in the first week of treatment at 28 mg/kg concentration, and the values fell rapidly on the second week along with the relapse of parasitemia.

Lymphocytes are the main effector cells of the immune system in mice [29]. Lymphocytosis has been implicated in trypanosomiasis and usually resulted from wax and wear syndrome in the animal immune system caused by the ever-changing variable surface glycoprotein of the infecting trypanosomes [30] which demands the immune system to continuously produce antibodies and hence keep the level of lymphocytes high. The present results of differential white blood cell counts revealed that both plant extracts decreased the percentage of lymphocytes compared with the negative controls in a dose-dependent manner. These findings are in agreement with those of the previous study done by Feyera et al. [20], which reported that plant extracts which decreased parasite load also decreased lymphocytosis and improved the immune system of mice by increasing the levels of other sets of white blood cells mainly neutrophils and monocytes.

## Conclusions

From the present study, it can be concluded that both plant extracts can reduce the level of parasitemia, improve body weight and prevent anaemia in a dose-dependent manner suggesting their antitrypanosomal activity. The study also showed *C. citratus* leaves had better activity than *L. sativum* seeds extract and even better than the standard drug of diminazene aceturate (DA) suggesting its high potential to be developed as an effective antitrypanosomal drug.

## Data Availability

All data included in the manuscript are the original data obtained from the research. The datasets generated and/or analysed during the current study are not publicly available due to the project CEVMed-TR is conducting a series of works using the current data as a baseline and all data will be public after the project  is completed but are available from the corresponding author on reasonable request.

## Abbreviations

DA: Diminazene aceturate
*C. citratus*: *Cymbopogon citratus*
*L.sativum*: *Lepidium sativum*
PCV: Packed cell volume
WBC: White blood cell
*T. congolense*: *Trypanosoma congolense*
AAT: Animal African Trypanosomiasis
HAT: Human African Trypanosomiasis
